# Indents

**Published:** 1903-08-15

**Authors:** 


### INDENTS.

☞ **Brief Articles of Technical or Scientific Value will receive notice and comment. Contributions invited.**

**Polishing Stains from Lingual Surfaces of Lower Incisors.** Frequently it will be found difficult to reach these surfaces with the ordinary rotary brushes. Substitute for these a moose-hide wheel about one-half inch in diameter, and run it as nearly as possible parallel with the long axis of the tooth. In this way the most inaccessible surfaces may usually be reached. The wheel, however, is not so effective for accessible surfaces as the brush.—*Dental Review.*

**Increase Standard of State Examination.** While we are increasing the college course to four years, making the curriculum more extensive, and the standard of graduation higher, it will naturally draw a great many who wish to enter the practice of dentistry to seek to enter the profession through the examining boards, thinking that perhaps they can pass the examining boards with less difficulty than they can procure a diploma at a dental college. So, it occurs to me, that while we are raising the standard of the colleges, we should endeavor to keep the standard required for passing an examining board on the same plane, else we might get the standard of the college course so high it would keep young men out of the schools and lead them to seek the other method of entering practice.—Dr. Prosser, in *Western Journal.*

**More Technical Work in the Four Years' Course.** The four years' course ought not to be based so much upon the theoretical instruction that may be given the student during that time as to afford them a longer time for the acquirement of the manual dexterity which is necessary to make a dentist. The time necessarily given to their medical studies, their scientific and theoretical studies, does not leave much time which can be devoted to purely manual practice. While men may have the natural instinct, it is hardly possible that dexterity can come by accident; it never comes except by long and continued practice. While these men come out from our colleges to-day fairly well equipped to begin practice, they really acquire most of their fitness to practice dentistry in the next two or three years at the expense of their patients. Our men ought to leave the college practically where they are after they now practice one or two or perhaps more years.—Dr. Wilmott, in *Western Journal.*

**Foreigners as Technickers.** These Old Country men are not good operators, with very rare exceptions, and I think that is to be accounted for wholly by considering the class of men from which dental students are drawn. Those of you who are acquainted with Old Country habits know that in certain circles manual labor is not looked upon exactly as a disgrace, but as shutting one out of a certain social circle; consequently, in certain social strata, men never earn their living by the work of their hands; they may be lawyers and bankers, but have no use for mechanical ability, and, naturally, after a course of years, if there has ever been any such mechanical training in their ancestors, it has been eliminated by want of development; consequently, men drawn from those social strata are not naturally qualified to make dentists; they have not that mechanical instinct, that mechanical taste, and unless at least the germ of it is there, no superstructure of skill can be built upon it.—Dr. J. B. Wilmott, in *Western Journal.*

**Wisconsin's New Law.** Its Examining Board consists of five members, three of whom shall be members of the State Dental Society. The State Society *may* nominate candidates. All persons licensed to practice must register annually and pay a fee of one dollar for so doing. After a written notice from the board, if a licensed practitioner fails to re-register in ninety days, his license may be forfeited by the Board. The board, in its discretion, may license without examination only graduates of colleges having a four years course of at least seven months each and requiring at least two years of high-school work for admission, except; that students attending Wisconsin colleges may be licensed providing they continue their studies until graduation. An unlicensed practitioner may be fined from ten to one hundred dollars for each patient treated. Practicing dentistry in Wisconsin is defined as treating diseases or lesions of the human jaws or teeth, or performing operations thereon, to *manufacture* or insert any artificial teeth, fixtures or appliances for the restoration, regulation or improvement of the dental organs, except by students in college and physicians regular work.

The examination fee is ten dollars.

**Bromo-chloron.** The copyrighted name for a salt of hypobromo-chlorite of lime, which is readily soluble in water. It is claimed to be non-irritant and non-toxic.

Last summer a gentleman while absent in the country had the misfortune to have the pulp in the lower left bicuspid die. He abandoned his vacation and came home, went to his physician, who lanced the abscess, which by that time had fully developed. A week later I saw him, and found the swelling somewhat subsided, and the abscess freely discharging pus. After opening and cleaning the root-canal with bromo-chloron, I flushed the abscess thoroughly through the fistula with bromo-chloron. I discharged several syringefuls into it, until not a trace of pus remained. (I may remark in passing that no precautions were taken to prevent the bromo-chloron from flooding the mouth, and, in fact, it did flood the mouth thoroughly.) At the next visit a week later, the abscess had completely disappeared; the root and tooth were then filled and the patient dismissed—

that is, at the second visit, the tooth and abscess having been treated but once. It may be that this was an exceptionally favorable case, because of the recent formation of the abscess, but even so, I think you will agree with me that it was a very quick cure.—D. W. Barker, in *International.*

**Good Advice.** The following excerpt taken from a paper published in the June *Cosmos*, by Dr. John T. Metcalf, of New York City, who was the first to publish in 1861 a formula for making an oxy-chloride of zinc filling material, is worth keeping and may well be copied into the written rules of every young man entering the dental profession:

"And now, gentlemen, after sixty-four years of experience and observation along dental lines, perhaps you will pardon the natural tendency of an octogenarian to moralize. Advice is always a cheap commodity, yet I feel that you of the younger set will pardon me, and agree with we, when I emphasize the necessity of looking well to the esthetic side of practice. Be neat at all times, almost to fastidiousness, as well as in dress as in speech and in office paraphernalia. On the prophylactic side let me say, Be careful about antisepsis; and at the chair be thorough and generous, looking always to the interest of the patient before the ultimate dollar—a well-established habit in this direction being on the side of love and mercy, and insuring a sound conscience, sound sleep, and a healthy, well-earned appetite for the good and true things of life. Toward your brother dentist be cordial, and free to share your discoveries and favors, avoiding unseemly boasting; and lastly, set your faces like flint against evil-speaking, and throttle scandal as you would throttle a crawling viper."

**Making a Dentist.** I would like to speak on one point that has been referred to in the discussion by one of the speakers. It is a very important point, too, in connection with this matter of education, and we should strive to understand it. It requires four years to make a machinist, and during these four years he spends all his time in the machine shop, doing only practical work, so that he may become able to turn a journal to a nicety, cut a toothed wheel perfectly, adjust his machines perfectly and do excellent mechanical work. But if this man has not during this time been studying his mathematics and his physics, or the laws of motion and mechanical drawing, and all those things, he will be simply a mechanic to be directed by the master—nothing more.

The dental colleges may make men who will fill a cavity well; they may produce men who will make a plate very well, indeed, but they are like the mechanic who has stuck to his machine shop. They are men who can work only when directed by others who have had a superior education along the lines relating to the specialty they fell into in dentistry. We should so equip each man that he will be a master, and until we do that we are not making the dentist we wish to send out to practice. Of course, we do not expect to do all this in four years, but we must give him the basis upon which to work in the future in his practice that he may work himself up to this point.—Dr. Black, in *Review*.

**Bridge-work.** That a man must eliminate his personality, and work along one line, is a practical impossibility. I cannot comprehend where men get the idea that the pulp must be destroyed in every tooth that is crowned. They remind me of the men who run down bridge-work and say, "You destroy the living tissue when you crown a tooth," but advocate the use of forceps to prepare the same mouth for a plate. They decry bridge-work because in some cases there is resulting filth, but the same men will put in a rubber plate, which is the filthiest thing in the world. Why is it that the man who advocates removal of the pulp in crowning operations will insert a large contour filling which contains more gold than crown? The thermal action is greater in that filling than in the crown, because it is closer to the pulp. The same men will advocate a porcelain filling to avoid a show of gold. It is not the gold upon the surface of the tooth that kills the pulp, but the phosphoric acid in the cement, and why would the acid not have the same effect when used to set a large porcelain filling? I do not believe that we should bind ourselves to any one method in crown and bridge-work. Our work should be an art as far as possible, but our first duty is to give our patients the greatest amount of satisfaction and the best service. When necessary we may sacrifice art for strength and utility. I have no faith in mere gingerbread work, but believe in putting something in the mouth that will remain there.—Dr. E. A. Bryant, in *Digest.*

**Artificial Dentures.** In the making of an artificial denture, the three principal points to be considered are: First, restoration of the lost function of mastication; second, restoration of the partially lost power of distinct articulation; third, restoration or preservation of the profile and expression of the face. Allowances must be made for the absorption of muscles of the mouth after the teeth are extracted, and also for the shrinkage of the arch. This is one great fault with artificial dentures, the patient's face presenting in many cases a shrunken appearance suggesting old age. Another great fault is "too much teeth." The patient usually insists upon a much smaller tooth in the artificial denture than they had when they wore their own teeth.

There are six faults that are the most general in artificial dentures, as follows: The teeth are too small; they are too light in color; too perfect in alignment; not adapted to the age of the patient; muscles of expression not sufficiently supported; expression injured by too close an occlusion. The first four faults are frequently the fault of the patient, who wants small white teeth, and wants them set perfectly. The average dentist follows too closely the "ideal perfect" in making his case, and as a result his denture shows plainly that it is artificial. We should follow as closely as possible the patient's own mouth and teeth, if the teeth are in the mouth when the case is taken. Strive to preserve as far as possible the original expression. The "ideal perfect" mouth is too rare to use as a model.—Dr. J. B. Willmott, in *Digest*.

**Don'ts.** Don't be careless; nothing is ever "near enough" in bridge-work; it most be absolutely correct.

Do not make your fee for bridge-work at a rate of "so much per tooth."

Do not use low-grade solder on a crown or bridge. It contracts, pits, oxidizes, and makes weak construction.

Do not forget that gargling the mouth and throat with a solution of carbolic acid accomplishes the same purpose as swabbing with hydrochlorate of cocaine solution in cases where the impression material is inclined to cause gagging.

Do not take out the impression when taken in compound, until you are positive it has thoroughly hardened and cannot change its shape. When an impression will not come out when thoroughly hardened, as in some partials, use plaster of Paris, and break off before it has become too hard, uniting the pieces afterward.

In taking plaster impressions of partial cases, do not forget to vaseline the teeth.

In filling with chloro-percha do not use more eucalyptus or other essential oil than will just moisten the canals, and don't use the chloro-percha in thinner solution than necessary to work into the canal. Keep gutta-percha points sterile in boracic acid.

Don't fill all root-canals with mummifying paste.

Whatever you use for the root-canal filling don't fail to fill the pulp chamber with oxychloride or oxyphosphate to prevent discoloration.

Don't attribute unworthy motives to another practitioner because he joins some society, or because he attends church. The man who emits this variety of "hot air" usually spends Sunday at the operating chair. He finds it pays better.—*Dominion Journal*.

**Heterogenesis.** The human mind has a tendency to swing from one extreme to another, and we are apt to think that a single new fact disproves everything previous. On the contrary, in the scientific world there are few revolutions and there is a continuity in the development of thought. Each fact discovered takes its harmonious place with what has preceded it. Heterogenesis as a biological fact was disproven long ago. There is nothing more fundamental in biology to-day than the universal law of heredity, namely, that all living things are derived from similar living parents. A correlative with this fact is the law of variation—that no living thing exactly resembles either of its parents. Up to the last decade the study of bacteriology was concerned with the identification of species and their relation to certain diseases, which latter has been proven. In the future we shall not overthrow those beliefs, but will study the modification of the species by its environment. Bacteria resemble other organized things in that their environment affects their functions. Biologists to-day recognize that all the phenomena of life presented through the cellular organization are but exalted chemical reactions, and that these reactions are dependent upon the reagents present. By changing the food of a poisonous snake its secretions may cease to be venomous, but give it again its proper food, restore its natural environment—and its bite will be as poisonous as ever. The same thought applies in bacteriology. Certain germs produce given diseases under certain conditions, but not under others, but it can not be proven that tetanus bacilli can produce tuberculosis. The same disease may be produced by different germs, and the same germs, may, under varying conditions produce what are recognized as two diseases, for instance, croup and diphtheria pulmonary pneumonia and pericarditis, but that does not annihilate the identity of species nor establish heterogenesis in biology.—F. B. Noyes, in *Digest*.

